# Nanoparticle-Based Assays for Antioxidant Capacity Determination

**DOI:** 10.3390/antiox14121506

**Published:** 2025-12-15

**Authors:** Jolanta Flieger, Natalia Żuk, Ewelina Grabias-Blicharz, Piotr Puźniak, Wojciech Flieger

**Affiliations:** 1 Department of Analytical Chemistry, Medical University of Lublin, Chodźki 4A, 20-093 Lublin, Poland; 2 Department of Plastic Surgery, St. John’s Cancer Center, Jaczewskiego 7, 20-090 Lublin, Poland; 3 Institute of Health Sciences, John Paul II Catholic University of Lublin, Konstantynów 1 H, 20-708 Lublin, Poland

**Keywords:** gold nanoparticles, cerium oxide nanoparticles, silver nanoparticles, metal nanoparticles, metal oxide nanoparticles, antioxidant activity/capacity, Localized Surface Plasmon Resonance (LSPR), sensors, nanostructured sensors, nanozymes

## Abstract

Thanks to both endogenous and exogenous antioxidants (AOs), the antioxidant defense system ensures redox homeostasis, which is crucial for protecting the body from oxidative stress and maintaining overall health. The food industry also exploits the antioxidant properties to prevent or delay the oxidation of other molecules during processing and storage. There are many classical methods for assessing antioxidant capacity/activity, which are based on mechanisms such as hydrogen atom transfer (HAT), single electron transfer (SET), electron transfer with proton conjugation (HAT/SET mixed mode assays) or the chelation of selected transition metal ions (e.g., Fe^2+^ or Cu^1+^). The antioxidant capacity (AOxC) index value can be expressed in terms of standard AOs (e.g., Trolox or ascorbic acid) equivalents, enabling different products to be compared. However, there is currently no standardized method for measuring AOxC. Nanoparticle sensors offer a new approach to assessing antioxidant status and can be used to analyze environmental samples, plant extracts, foodstuffs, dietary supplements and clinical samples. This review summarizes the available information on nanoparticle sensors as tools for assessing antioxidant status. Particular attention has been paid to nanoparticles (with a size of less than 100 nm), including silver (AgNPs), gold (AuNPs), cerium oxide (CeONPs) and other metal oxide nanoparticles, as well as nanozymes. Nanozymes belong to an advanced class of nanomaterials that mimic natural enzymes due to their catalytic properties and constitute a novel signal transduction strategy in colorimetric and absorption sensors based on the localized surface plasmon resonance (LSPR) band. Other potential AOxC sensors include quantum dots (QDs, <10 nm), which are particularly useful for the sensitive detection of specific antioxidants (e.g., GSH, AA and baicalein) and can achieve very good limits of detection (LOD). QDs and metallic nanoparticles (MNPs) operate on different principles to evaluate AOxC. MNPs rely on optical changes resulting from LSPR, which are monitored as changes in color or absorbance during synthesis, growth or aggregation. QDs, on the other hand, primarily utilize changes in fluorescence. This review aims to demonstrate that, thanks to its simplicity, speed, small sample volumes and relatively inexpensive instrumentation, nanoparticle-based AOxC assessment is a useful alternative to classical approaches and can be tailored to the desired aim and analytes.

## 1. Introduction

**Figure 1:**
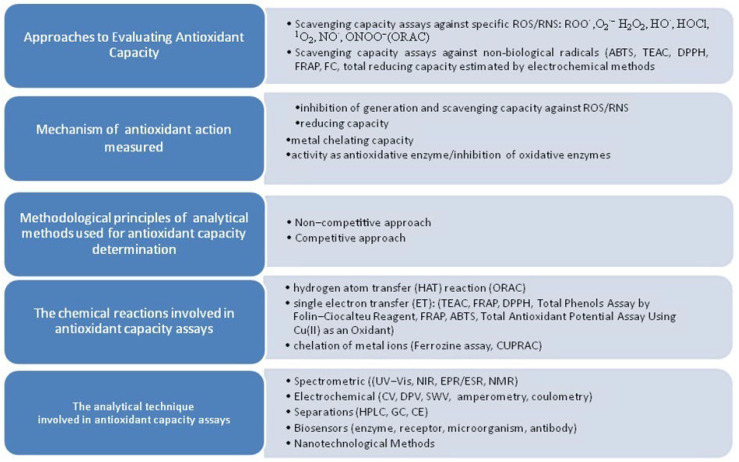
Classification of AOxC methods into categories according to selected criteria.

An imbalance between the oxidative and antioxidant systems can lead to an increase in the production of reactive oxygen/nitrogen species (ROS/RNS), which play an important role in the development of many diseases [1]. Natural dietary antioxidants (AOs), such as vitamins, carotenoids, polyphenolic compounds and flavonoids, can reduce oxidative stress and slow the development of diseases by delaying or preventing oxidation [2]. Several methods have been developed to determine the antioxidant capacity (AOxC) of various samples, including supplements, foods, and plant extracts [3,4]. These approaches can be categorized depending on the criterion applied. Figure 1 presents the selected criteria for characterizing AOxC tests [5].

In addition to UV-vis, fluorescence and electrochemical techniques, other techniques are gaining popularity in the study of AOxC, such as electron paramagnetic resonance (EPR) spectroscopy [6,7,8]. The EPR method enables direct measurement of free radical concentrations, and analysis of EPR spectra allows identification of the chemical nature of the observed paramagnetic species. EPR spectroscopy is particularly effective at detecting hydroxyl (OH^•^), superoxide (O_2_^•−^) and nitric oxide (NO^•^) radicals. As the intensity of the EPR signal is dependent on the concentration of free radicals, determining antioxidant properties involves measuring the EPR signal of stable radicals such as 1,1-diphenyl-2-picrylhydrazyl (DPPH), 4-hydroxy-2,2,6,6-tetramethyl-1-piperidinyloxyl (TEMPOL), and galvinoxyl, following their interaction with antioxidant compounds. EPR can also be used to study magnetic nanostructures and their modifications [9]. For example, this technique can be used to determine the type of magnetic iron oxide nanoparticles (Fe_3_O_4_, γ-Fe_2_O_3_, α-Fe_2_O_3_, or combinations thereof). The technique’s capabilities are further expanded by spin labeling polymer-coated nanoparticles, enabling the study of their interactions with biological fluids. In a study by Prima et al. [10], EPR coupled with spin trapping was used to evaluate the antioxidant properties of vegetable oils that produced free radicals following UV irradiation. N-tert-butyl-α-phenylnitrone (PBN) was used as a spin-trapping agent that forms stable, long-lived nitroxide spin adducts with highly reactive, short-lived free radicals, such as hydroxyl or lipid radicals.

Nanotechnology offers a different approach to assessing AOxC. Nanomaterials (with at least one dimension of less than 100 nm) have unique optical and catalytic properties (Surface Plasmon Resonance—SPR), which can be utilized for quantification. They can therefore be used as sensors to determine AOxC with various spectrometric and electrochemical detection systems. It should be emphasized that the optical, mechanical, electrical and magnetic properties of nanoparticles differ significantly from those of microparticles and bulk materials [11,12,13,14,15,16,17,18] and can be controlled by adjusting their size, shape and degree of agglomeration [19].

Nanoparticle-based assays (including AuNPs and AgNPs) fall under the category of non-radical, redox potential-based assays and typically operate via an electron transfer (ET) mechanism. Chemical reduction-based nanotechnological assays for colorimetric AOxC measurements exploit the generation, growth or aggregation of nanoparticles (NPs), primarily noble metals (e.g., AuNPs and AgNPs), following the reaction of their ions with an antioxidant (AO) [20,21]. Pure nanomaterials have low molecular specificity and cannot distinguish between individual analytes. However, nanoparticles, particularly AuNPs, can be used as modifiers for electrochemical sensors, improving sensitivity and detection limits [22]. Adding nanomaterials to electrochemical sensors increases the surface area for immobilizing sensing molecules (e.g., antibodies, enzymes and DNA) and boosts electron transfer kinetics. A significant advance in AOxC evaluation is the use of nanomaterials with catalytic activity similar to that of natural enzymes, known as nanozymes, which offer specific selectivity [23]. A milestone in AOxC testing is the use of nanomaterials with an ultra-small size (less than 1 nm), including carbon dots (CDs) and graphene quantum dots (GQDs), which have unique fluorescence properties and are biocompatible [24]. These materials can be produced from waste and offer multimodal platforms as fluorescent and colorimetric probes, as well as catalysts.

In recent years, numerous reviews have been published on methods for assessing antioxidant capacity and activity. In many of these reviews, nanosensors are mentioned in a single subsection. Between 2005 and 2025, a few reviews in the PubMed database were devoted to nanosensors for AOxC testing [17,18,25,26,27,28,29,30,31,32,33]. Some focus solely on electrochemical studies, others on fluorescence detection, others on luminescent quantum dots, and others on single metallic nanoparticles, such as AgNPs.

In antioxidant studies, nanomaterials (e.g., quantum dots (QDs)) can act as sensors that react with free radicals by changing color, fluorescence or conductivity. Metallic nanoparticles (AuNPs, AgNPs) and metal oxide nanoparticles (ZnO, TiO_2_) act as LSPR-based sensors, changing their LSPR colors/peaks when interacting with antioxidants. These can be used to create advanced platforms for the rapid, parallel evaluation of multiple antioxidants (high-throughput screening). There are numerous types of nanomaterial with different structures and various applications, including in cosmetics [34], industry and agriculture [35,36], and medicine [37,38]. This review focuses on nanomaterials known to be effective in AOxC tests, particularly metallic nanomaterials and QDs (semiconductor nanocrystals, graphene and carbon quantum dots), and presents key findings regarding their use. Particular attention is paid to the methodologies and mechanisms of the tests developed for this purpose. In line with this goal, the following sections will provide a critical review of the available literature on this topic.

## 2. Localized Surface Plasmon Resonance (LSPR)

A feature of metallic nanoparticles used in AOxC measurements is the phenomenon of localized surface plasmon resonance (LSPR), which causes the emission of light in the visible range, enabling colorimetric measurements. LSPR occurs when conduction electrons collectively oscillate under the influence of electromagnetic radiation of the same frequency [39,40,41].

The strong absorption band in the visible light range resulting from LSPR at a specific wavelength is used for quantitative detection. The light absorption capacity depends on the shape and size of the nanoparticles, as well as intermolecular interactions and the refractive index of the dispersion medium [42,43,44,45,46]. Since stronger antioxidants can form smaller NPs and the LSPR depends on particle size (smaller NPs = blue-shift, larger = red-shift), a significant shift in the maximum wavelength (λmax) may occur with changes in antioxidant strength, making quantification difficult [29]. Kinetic issues are resolved by controlled enlargement (increase in NP size) of nanoparticles, e.g., AgNPs. The Silver Nanoparticle Antioxidant Capacity (SNPAC) method utilizes the addition of Ag seed nanoparticles, which are coated with newly formed NP layers under the influence of polyphenolic antioxidants.

LSPR is a nanoscale phenomenon, mainly related to the nanostructures of noble metals such as gold (Au) and silver (Ag) [47]. In addition to noble metals, other metals such as aluminum (Al) and copper (Cu) also exhibit the LSPR phenomenon [18,31,33,48,49,50].

Plasmon oscillations are spatially confined in particles of sizes on the scale of light wavelengths, namely nanoparticles (NPs). These oscillations do not propagate over longer distances along the surface; hence, LSPR exhibits a non-propagating character in contrast to propagating plasmons, which spread out and occur on flat surfaces or continuous thin metal layers, known as surface plasmons (SP) [47,51].

The most important consequence of the non-propagating and localized nature of LSPR is the generation of a short electromagnetic (EM) field decay length in close proximity to the nanoparticle surface. The electromagnetic (EM) field near the nanoparticle surface, resulting from LSPR, rapidly diminishes with increasing distance. This short decay range (the so-called decay length) is typically about 10 to 30 nm.

Other resonant effects include optical extinction. The peak of optical extinction (representing maximum absorption and scattering) is visible in noble metal NPs at the resonance frequency [47].

The LSPR phenomenon is sensitive to environmental changes [51]. When molecules bind to a surface, a change in plasmon resonance occurs, which is detected as an altered plasmon signal, for example, as a shift in the LSPR spectral peak [52]. The position of the LSPR quenching (absorption/scattering) peak depends on the refractive index of the surrounding dielectric medium [53]. A change in the ambient refractive index causes a shift in the wavelength of the LSPR peak. The sensitivity and properties of LSPR depend on several factors, including the material (Ag generates sharper resonances and greater sensitivity), particle size (absorption dominates in smaller particles, scattering dominates in larger ones; for example, in gold nanospheres the transition from absorption to scattering dominance occurs at a diameter of about 80 nm), shape and aspect ratio (sharp shapes exhibit higher refractive index sensitivity, and nanorods have greater sensitivity and a redshift of the resonance peak) [47,54]. Furthermore, interactions between particles (aggregates) enhance LSPR effects. Aggregation is visible as a shift in the absorption spectrum toward longer wavelengths—a redshift [55].

To study antioxidant capacity, a band is used, which is attributed to the collective excitation of an electron gas, accompanied by a periodic change in electron density at the surface [56].

Strong absorption of visible light at a specific wavelength is the result of plasmon resonance absorption in MNPs. For individual metals, LSPR causes the appearance of surface plasmon absorption bands with characteristic intensity and location. In addition to the metal type, the color and optical properties of the nanoparticles depend on the shape, size, adsorbed molecules, and the refractive index of the dispersion medium. For example, spherical Au nanoparticles exhibit absorption in the visible spectrum (VIS) around 530 nm. In the case of nanotube-shaped particles, the resonance is significantly redshifted toward longer wavelengths. Silver nanoparticles (AgNPs) exhibit a strong absorption band (423 nm), which is absent in the spectrum of the bulk metal due to surface plasmon resonance (SPR).

AgNPs and AuNPs are most commonly used to study AOxC. AuNPs are utilized as metallic sensors due to their chemical stability and resistance to oxidation, while AgNPs provide sharper resonances and increased sensitivity. AgNPs are characterized by very high molar extinction coefficients (ε ≈ 3 × 10^11^ M^−1^ cm^−1^), and are expected to provide higher sensitivity in optical detection methods than conventional reagents [57,58]. However, it should be noted that the reaction sensitivity is also influenced by the reaction stoichiometry, particle size and shape, and dielectric constants, as well as their surrounding environment [59].

Optical sensors can operate using two fundamental strategies: aggregation/dispersion and non-aggregation (etching, growth) [60,61]. In aggregation-based sensors, the analyte induces NPs aggregation, which leads to plasmon coupling and a shift in the LSPR band (often accompanied by a change in solution color). Consequently, the analyte concentration is directly correlated with the degree of nanoparticle aggregation and the resulting change in the LSPR band. The dispersion (anti-aggregation) mechanism occurs when the colloid is initially aggregated, and the analyte acts as a dispersant, displacing the ligand that caused aggregation from the NP surface. In these systems, the analyte concentration is inversely proportional to the degree of NP aggregation. An example is the detection of methionine (Met) based on the anti-aggregation mechanism of gold nanoparticles (AuNPs) treated with melamine (Mel) used as an aggregating agent [62]. Non-aggregation strategies, based on etching and growth of metallic nanoparticles, modify the LSPR band by changing the size, shape, and dielectric environment of the NPs. Etching involves the catalytic oxidation of metallic nanoparticles, induced directly or indirectly by the analyte, which changes their size, shape, or composition and consequently modifies the LSPR band (an example is the oxidative etching of AuNTPs by Cr(VI)) [63]. Growth involves the generation of new plasmonic nanostructures from metallic ions, leading to the formation or rearrangement of the LSPR band and associated color changes in the solution.

Optical sensors based on the LSPR effect, utilizing metallic nanomaterials (MNPs), form two main categories of analytical techniques. The first category encompasses surface-enhanced spectroscopies (SES) [64]. SES techniques utilize the interaction of analytes with intense electromagnetic (EM) fields, which are generated and localized near nanoparticles (NPs) due to the LSPR effect. SES includes: (i) Surface-enhanced Raman spectroscopy (SERS): This demonstrates the most advanced level of performance compared to SEF and SEIRA. SERS achieves significantly higher enhancement factors (EF), increasing by six to ten orders of magnitude, which can lead to single-molecule sensitivity; (ii) Surface-enhanced fluorescence (SEF); (iii) Surface-enhanced infrared absorption (SEIRA): Similarly to SEF, this technique demonstrates a moderate increase in analytical signal, up to about two orders of magnitude, although the fundamental challenge for SEIRA is achieving reliable quantification. The second group consists of optical sensors based on LSPR band extinction (absorption/colorimetry). These sensors rely on monitoring visible light absorption, which is modulated by changes in the dielectric environment surrounding the NPs. These changes are the result of nanoparticle aggregation/dispersion mechanisms or non-aggregation strategies (such as etching or MNP growth).

## 3. Determination of Antioxidant Capacity/Activity Using Metallic Nanoparticles

Assays based on NPs for determining AOxC utilize LSPR for colorimetric detection. As reductants, AOs reduce metal salts to NPs and/or act as catalysts for nanostructure enlargement [46]. Several approaches are used to assess AOxC: (i) nanoparticle aggregation; (ii) nanoparticle seed enlargement in the presence of an antioxidant compound; and (iii) nanoparticle formation through salt reduction by AOs [65].

In addition to AgNPs and AuNPs, in vitro assays for measuring AOxC use iron oxide nanoparticles (IONPs), titanium oxide nanoparticles (TiO_2_NPs), titanyl oxalate, rhodium nanoparticles (RhNPs), zirconium oxide (ZrO_2_), zinc oxide (ZnO) and cerium oxide (CeONPs) by optically monitoring changes in localized surface plasmon resonance (LSPR) [57,66,67,68,69,70].

NP formation can usually be observed visually due to its characteristic color, which depends on the nanoparticle morphology, adsorbed ligands and the properties of the dispersion medium [71].

The main instrumental techniques used to analyze nanoparticles include: microscopic techniques (SEM, TEM and AFM, i.e., scanning electron microscopy, transmission electron microscopy and atomic force microscopy), which are useful for identifying morphological surfaces; X-ray photoelectron spectroscopy and energy dispersive X-ray spectrometry, which are useful for confirming elemental composition; X-ray diffraction (XRD), which is useful for determining the crystallography of NPs; UV–Vis absorption spectroscopy, with other spectroscopic techniques (FTIR, spectrophotometry, Raman spectroscopy, surface-enhanced Raman scattering (SERS) and surface plasmon resonance (SPR) spectroscopy) [72]; Dynamic light scattering (DLS) measures the size distribution, surface charge (or zeta potential), hydrodynamic radius and concentration of nanoparticles dispersed in solution.

Nanoparticle-based assays are usually calibrated using reference antioxidants [65,73,74,75]. Some authors report good agreement between total antioxidant capacity (TAC) results obtained using nanoparticle-based assays and those obtained using reference methods such as ORAC, TEAC and CUPRAC [76], as well as Folin–Ciocalteu, FRAP and DPPH [73]. Table 1 summarizes information regarding metallic and metal oxide nanoparticles that are used to determine antioxidant capacity and which employ LSPR-based measurements.

Reducing agents are required for producing metallic nanoparticles and inducing changes in existing NPs. To create MNP colloidal dispersions, sodium citrate, hydrogen peroxide and hydrides are typically employed as reducing agents [19]. The morphological characteristics of NPs depend on the reducing agents, dispersion stabilizers, and pH. Strong reducing agents react rapidly with inorganic salt or metal complex precursors (e.g., Ni, Co, HAuCl_4_, AgNO_3_, H_2_PtCl_6_, RhCl_3_, PdCl_2_), resulting in small NPs being formed [16]. Conversely, the reaction is slow for weak reducing agents, and the synthesized NPs are usually larger. In multicomponent samples such as plant extracts, all antioxidants participate in the formation of nanoparticles as the process is not selective [81]. Therefore, the kinetics of the reduction process depend on the total antioxidant capacity (TAC) of the sample. Consequently, the process of nanoparticle formation/growth can be used to assess TAC.

### 3.1. Silver NanoParticle Antioxidant Capacity (SNPAC)

In 2012, Özyürek et al. [56] used an AgNP-based method to evaluate the antioxidant properties of polyphenols. They used the absorbance band of the fabricated core–shell Ag NP structures at 423 nm for quantitative analysis. This method is known as Silver NanoParticle Antioxidant Capacity (SNPAC).

The SNPAC mechanism of action is based on electron transfer (ET). Unlike classical reduction methods (e.g., CUPRAC and FRAP), which directly reduce the metal-ligand complex, the SNPAC method utilizes the growth of silver nanoparticles (AgNPs) under the influence of the addition of an antioxidant (e.g., polyphenols or vitamins) as a secondary reducing agent [82,83]. Unfortunately, using an antioxidant as the sole reducing agent did not produce a linear response [56]. The SNPAC assay, also known as ‘Seed-Mediated Growth’, employs a two-stage procedure. The first stage involves preparing monodisperse silver nanoparticle seeds by reducing them with a weak reducing agent, trisodium citrate:




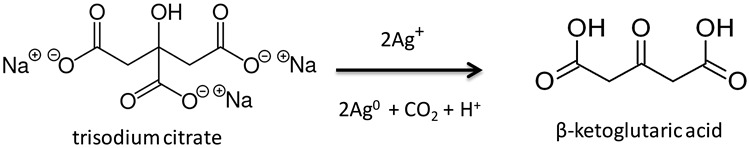

(1)



Citrate acts as both a reducing agent and a dispersion stabilizer, inhibiting the formation of new nucleation sites [84]. As a dispersion stabilizer, citrate imparts a negative charge to the nanoparticles, which causes electrostatic repulsion and enhances dispersion, thereby preventing aggregation. In the second stage of the test, the antioxidant (AO) being tested is added to the prepared nuclei and acts as a secondary reducing agent:

nAg^+^ + AO-(OH)_n_→nAg^0^(AgNPs) + AO(=O)_n_ + _n_H^+^ (2)

In the method proposed by Özyürek [56], the SNPAC reaction mixture consists of the following components, at a final volume of 2.8 mL: 2.0 mL of the initial AgNP seed solution; x mL of the standard antioxidant or actual sample solution; and (0.8 − x) mL of H_2_O. The most commonly used incubation time in SNPAC is 30 min, compared to 30–60 min in CUPRAC and 20–60 min in free radical-based methods (e.g., DPPH and ABTS). It should be noted that authors using conventional methodologies rarely consider the kinetics of free radical reactions, which may be slow for some antioxidants [4,85,86], potentially generating inconsistencies in the results obtained.

The E potential of Ag(I)/Ag° is 0.8 V, whereas the standard potential of most antioxidants is in the range of 0.2–0.6 V. Consequently, electrons are transferred from the antioxidants (at a lower potential) to the Ag^+^ ions on the surface of the nanoparticle seeds. This results in the seeds’ growth and the formation of a core–shell structure. Simultaneously, the phenolic groups are reduced to the corresponding quinones. An increase in antioxidant concentration results in a linear increase in LSPR absorption that is directly proportional and can be monitored spectrophotometrically at 423 nm. The intensity of the absorption band of AgNPs increases linearly with increasing antioxidant concentration. For polyphenols, high linear correlation coefficients (r^2^ = 0.994) and LOD values of approximately 1 nM were obtained [56]. The growth of AgNPs can be observed with the naked eye as the reaction mixture changes from light yellow to brown as the antioxidant concentration increases.

Total antioxidant capacity (TAC) is determined by the increase in absorbance (ΔA) at 423 nm, measured relative to the blank sample after 30 min of incubation at 25 °C following the addition of the antioxidant to seeded AgNPs. Absorbance increases linearly with increasing concentrations of different (hydrophilic and lipophilic) antioxidants. The TEAC of a given antioxidant is the ratio of its molar absorbance (ε) to the molar absorbance of Trolox, measured under the same conditions. Ozyürek et al. showed that, in the case of a mixture containing different antioxidants, the absorbances are summative, confirming that the antioxidant capacities are additive [56].

Numerous examples of the practical application of the SNAPC method to analyze complex samples in the form of natural extracts can be found in the literature.

This method has been employed to evaluate the antioxidant capacity of food and beverage samples, including fruit juices and herbal teas [56,87]. For example, Szydlowska-Czerniak et al. used a method based on AgNPs to determine the AOxC of fats and oils from various rapeseed varieties and rapeseed processing by-products, such as flakes and oilcakes [73]. Other authors have studied hydrophilic and lipophilic compounds such as polyphenols (flavonoids, simple phenolic acids and hydroxycinnamic acids) and vitamins C and E using SNPAC methodology [77,88,89,90,91,92]. Bukovsky-Reyes et al. used a modified SNPAC assay to determine the total antioxidant capacity (TAC) of distilled spirits, including whisky, bourbon, tequila and rum [93].

In pioneering work, Özyürek et al. [56] used silver nucleating particles obtained by reducing Ag^+^ ions with trisodium citrate as a weak reducing agent. In this method, 50 mL of 1.0 mM AgNO_3_ is heated to boiling point for 10 min. Then, 5 mL of 1% trisodium citrate was added dropwise and the solution stirred. When the color of the mixture turned light yellow, it was cooled to room temperature. In the work presented by Bukovsky-Reyes et al., 15 mL of an Ag^+^ solution with a concentration of approximately 500 mg/L (4.6 mM) was added to a 35 mL microwave vessel containing ~150 mg of soluble starch. The AgNPs were synthesized in a microwave oven at 150 °C for 15 min and pre-capped with starch instead of citrate. Using microwave heating, the incubation time was shortened from 30 min to 6 min, and the volume of the AgNP reagent was reduced from 2 mL to 0.5 mL.

The SNPAC method produces results that correlate with those obtained using other ion-reduction methods, such as the CUPRAC (Cupric Reducing Antioxidant Capacity) test [94]. TEAC values obtained using the SNPAC method were found to be comparable to those obtained using the CUPRAC test (for the 15 antioxidants tested, the correlation coefficient was 0.936). However, a slightly poorer correlation was observed between AgNP and FRAP, DPPH and total phenolic content (Folin–Ciocalteu) in different rapeseed extracts, including flakes, press cake and meal, with r values ranging from 0.5971 to 0.9149 [73,87].

Bukovsky-Reyes et al. [93] evaluated the SNPAC assay’s ability to respond to antioxidants with different reducing powers by preparing calibration curves for individual antioxidants and comparing the resulting slopes (ε) and gallic acid equivalents (GAE) (in mg/L and µM). These results were then compared with the reactivity of various compounds using the Folin–Ciocalteu reagent, as described by Everette et al. [95], as well as with the data provided by Özyürek [56]. Modest consistency was found between the two sets of data for the AOs collected in this study. The received molar GAE values were approximately half the reported values of Everette et al. [95]. However, it was noted that the standards used for the SNPAC assay were prepared in a water–ethanol mixture (60:40, *v*/*v*). Ethanol may have a prooxidant effect, which could account for the lower molar concentration ratio of GAE. To test this hypothesis, ellagic acid and gallic acid standards were prepared in deionized water and reanalyzed using the SNPAC assay. The resulting data were much closer.

Bukovsky-Reyes et al. [93] used HPLC to validate the SNPAC assay. As gallic and ellagic acid concentrations were the major contributors to the enhanced plasmon resonance of the AgNPs, these acids were used to validate the SNPAC assay measurements. They evaluated the correlation between HPLC and SNPAC assays by determining t-test values (assuming a normal distribution) and the Wilcoxon signed-rank test for a non-normal distribution. To assess the agreement between the HPLC data and SNPAC assay values, Bland–Altman plots were also used. Only two of the 33 samples included in this study were beyond the mean difference ± 2 SD values, indicating agreement between the two methods.

The advantage of the SNPAC method is its high colorimetric sensitivity, expressed by high molar extinction coefficients (ε ≈ 3 × 10^11^ M^−1^ cm^−1^) and the absence of interference from common substances present in food extracts, such as simple sugars (e.g., glucose, fructose) and amino acids (e.g., glycine, alanine), or components such as oxalates, citrates, and fruit acids [56]. This distinguishes SNPAC from the Folin–Ciocalteu method (FC) and nanoparticle-based assays, where non-phenolic compounds such as ascorbic acid, aromatic amines, and metal ions interfere with measurements [56], potentially leading to inaccurate test results. Mechanism-based (ET) methods require appropriate pH conditions to ensure optimal sensitivity. In the case of FRAP, an acidic environment (pH 3.6) is preferred [96]. For CUPRAC, pH 7 is optimal [97], whereas SNPAC provides the most favorable absorbance response at pH 6.0 [56].

A major advantage of the SNPAC method is its selectivity and the linear dependence of absorbance on concentration over a wide range of concentrations, which allows for comparison of TAC values across different samples. The two-step procedure and preparation of seed NPs ensure a wide linear range for the method. Furthermore, SNPAC (like modified CUPRAC or ORAC assays) can measure the TAC of both hydrophilic and lipophilic antioxidants (e.g., vitamins C and E and polyphenols).

“Turn-off” nanoparticle probes, which operate based on the disintegration of NPs, can be used to measure TAC through their ability to scavenge ROS [98,99,100]. An example is LSPR sensors, which operate by degrading AgNPs, catalyzing the decomposition of hydrogen peroxide, resulting in a visible discoloration of the solution. AOs, by scavenging hydrogen peroxide, inhibit this disintegration, restoring the LSPR signal. A disadvantage of this assay is the difficulty in obtaining a linear relationship between the signal and the antioxidant concentration.

### 3.2. Gold Nanoparticles (AuNPs)

Scampicchio et al. [77] were the first to describe a method for measuring antioxidant activity based on the catalytic growth of gold nanoparticles (AuNPs) under the influence of phenolic acids, which they published in 2006.

Using the method described by Enüstün and Turkevich in the 1960s, a stable dispersion of 20 nm AuNPs was obtained by reducing Au^3+^ salt with sodium citrate at 100 °C [101], or with other reductants such as borohydride, formaldehyde and stabilizing agents like thiol-functionalized organics, surfactants or polymers (e.g., heparin [102]). The use of AuNPs in TAC assays is based on redox reactions, whereby antioxidants reduce gold ions (Au^3+^) [78,101,103,104,105,106,107], resulting in the generation or growth of NPs [77,78,101,108]. The mechanism for determining TAC using AuNPs is based on electron transfer (ET). AuNPs can be detected using SERS or UV-visible spectroscopy due to their plasmonic properties. The absorption maximum of 10 nm AuNP nanoparticles occurs at 520 nm. A solution of well-dispersed AuNPs turns red. Aggregation and decreasing spacing between individual AuNPs shifts the LSPR absorption band towards longer wavelengths. Colloidal dispersions change color to various shades of violet-blue [109,110].

The antioxidant (AO) acts as an electron donor for the gold ions (Au^3+^), which are derived from the AuCl_4_^−^ ion and are reduced to metallic gold (Au^0^). The ET reaction can be written in general terms and for the example antioxidant, ascorbic acid (AA), as follows:

Au^3+^ + AO {reduced} ↔ Au^0^ (AgNPs) + AO {oxidized} (3)

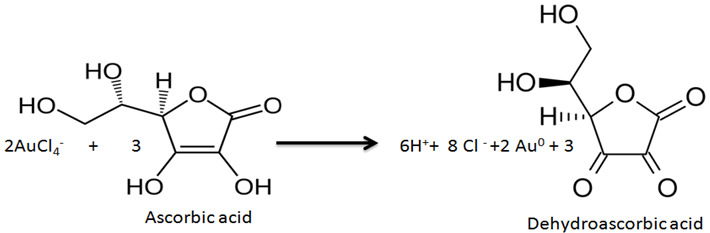

(4)



The monitoring of AuNPs is associated with a characteristic absorbance peak, typically around 517, 540 or 555 nm. The color of AuNPs depends on their shape and size, as well as intermolecular interactions and the refractive index of their environment.

Scampicchio et al. were the first to develop an AOxC assay that utilizes the generation and growth of AuNPs from a mixture of gold salts, citrate and cetyltrimethylammonium chloride (CTAC), when phenolic acids (vanillic acid, propyl gallate, protocatechuic acid, caffeic acid and ferulic acid) are added [77]. The localized surface plasmon resonance (LSPR) absorbance of the AuNPs, measured at 555 nm, was found to be linearly correlated with acid concentration. The plasmon absorption band was measured in a 0.01 M phosphate-buffer solution at pH 8.0 after an incubation period of 10 min at 45 °C. The authors compared the new Au NP protocol with total phenolic content determined by the FC test (r = 0.993) and cyclic voltammetry. Subsequent studies used the AuNP growth-based method to analyze phenolic acids and flavonoids in food samples such as tea, apples, pears, wine and honey [18,101,108,109], as well as selected isoflavone classes including aglycones (genistein and daidzein) and their glycosides (genistin and daidzin), soy extracts and the plants Tagetes lucida, Mentha piperita, Cynara scolymus, Cymbopogon citratus and Calendula officinalis. Polyphenol standards such as chlorogenic acid, rutin and hesperidin were also analyzed [111,112].

Wang et al. [111] studied the UV–vis absorption spectra of Au-NP seeds and enlarged NPs when treated with different concentrations of flavonoids, such as quercetin, daizeol and puerarin, which were present in the growth solution. They obtained a linear range with r = 0.9991–0.9999. They also employed an electrochemical technique to study the enlargement of the seeds of the gold nanoparticles (Au-NPs) onto a modified electrode by reducing tetrachloroauric acid using flavonoids as a source of gold.

Reza Hormozi Nezhad et al. [112] developed a colorimetric method for detecting a series of dihydroxybenzenes (DHBs) and their derivatives (e.g., hydroquinone, catechol, and pyrogallol). The reduction of AuCl_4_^−^ to AuNPs by AOs in the presence of CTAC produced LSPR absorbance at λ = 568 nm that was linearly correlated with the concentration of AOs.

In exploring AuNP synthesis as an analytical tool for AOxC evaluation, Escarpa’s group made an important observation [18,113]. The researchers described the synthesis of AuNPs using soy isoflavone extracts [113]. The dependence of absorbance at 540 nm on concentration was defined using a sigmoidal curve. Based on this curve, the AOxC concentration at which absorption reaches half-value was determined, as well as X_c_^50^_,_ which indicates the AuNP production efficiency, and K_AuNPs_, which states the amount of AuNPs produced per concentration unit. The synthesis of AuNPs of different sizes and colors was associated with the antioxidant activity of various isoflavones. Genistein and soy extracts with high aglycone content exhibited the highest antioxidant activity, producing the most intense red colloidal solutions and the smallest particle sizes. The authors emphasize the consistency of the Au NP protocol results with respect to X_50_c (the concentration value at which absorption reaches half-value) and total phenolic compounds (r = 0.93, *p*-value < 0.05), as determined by the Folin–Ciocalteu (FC) test, as well as with respect to FRAP and ABTS (r = 0.85, *p*-value < 0.10). The relationship between the concentration of antioxidants and LSPR absorbance was measured for four selected polyphenols: chlorogenic acid, and the flavonoids rutin, apigenin and hesperidin, which belong to the flavonol, flavone and flavanone classes, respectively. The obtained relationship was sigmoidal, described by the equation:

(5) $$A_{540}=\frac{A_{max}}{1+e^{-K}(X-X_{c}^{50})}$$

where A_max_ is the maximum absorbance at 540 nm, X is the polyphenol concentration, X_c_^50^ denotes the inflection point, and K is the factor fit with the slope units, which is the parameter expressing the AOxC.

AuNPs have also been used to study the antioxidant capacity of chrysanthemum extracts, various teas (black, green), fruit juices (orange), edible oils, including olive oil, and to detect adulteration of argan oil [69,77,78,106,114].

AuNPs are also key in electrochemical methods for determining antioxidant activity. Gold nanoparticles (the most commonly used) can serve as a conductive matrix in biosensors, increasing the surface area of the working electrode and thus leading to higher currents and increased sensitivity. Amperometric TAC measurements of plant extracts using the electrochemical Au-nanozyme sensor (based on the enzyme-like catalytic activity of AuNPs) showed good correlation with the chemiluminescence method (Pearson’s correlation coefficient of 0.958) [103].

**Figure 2:**
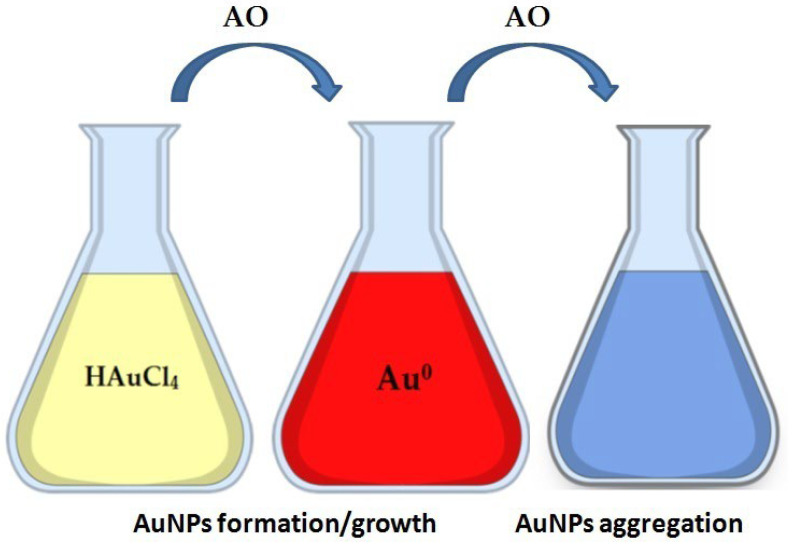
Main mechanisms and accompanying color changes in AuNPs used in antioxidant capacity assays.

Several strategies for using AuNPs for the quantification of AOs have been described in the literature, including (i) antioxidant-induced AuNP formation and growth (AuNPs formation/growth). Antioxidants (e.g., phenolic acids, vitamin C) reduce Au^3+^ ions or HAuCl_4_ to AuNPs and other products. In electrochemistry, the growth of AuNPs on the electrode surface is monitored using cyclic voltammetry (CV); (ii) nanoparticle growth (Enlargement). In this case, AOs act as catalysts for the enlargement of nanostructures (e.g., nanoshells). Precursor nanocomposites containing a SiO_2_ core with added AuNPs can serve as nucleation sites; the analyte (phenolic antioxidant) reduces AuCl_4_^−^ to Au^0^, inducing the growth of gold nanoshells on the SiO_2_ core [74,75,115,116,117,118]. The growth of AuNPs on the electrode surface was also monitored using CV; (iii) AuNP aggregation (AuNP aggregation). Some types of AOs, especially thiols (e.g., cysteine), can form a self-assembling layer on the AuNPs surface. This leads to nanoparticle aggregation and a shift in λmax to longer wavelengths (the batachromic effect) [119]. In this version, we observe a color change from red (dispersed nanoparticles) to blue (AuNPs aggregates) upon addition of an antioxidant. (Figure 2).

**Figure 3:**
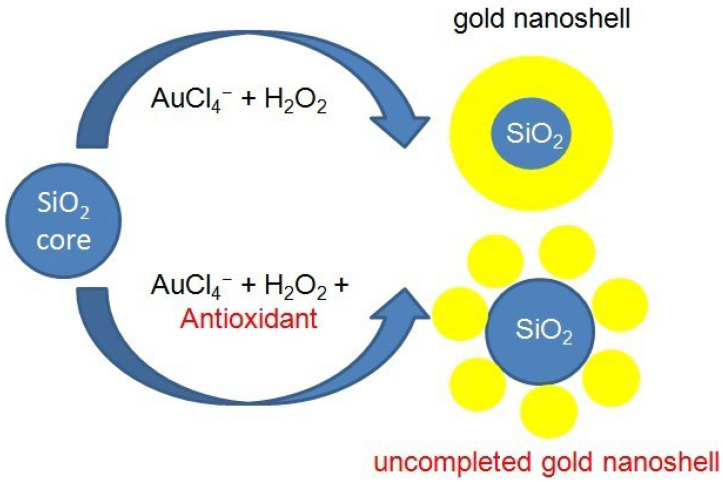
Antioxidant capacity assay utilizing AuNPs nanoshell growth inhibition.

Sudeep et al. [116] described a highly selective (in the presence of various other amino acids) and sensitive (3 µM cysteine, 12 µM glutathione) method for detecting the endogenous antioxidants cysteine (Cys) and glutathione (GSH) using Au-nanorods, which undergo aggregation, visible as an LSPR absorption band at 850 nm. Nanotube aggregation is closely related to the feature anisotropy and plasmon absorption coupling. The thiol functional groups of Cys and GSH in their zwitterionic form promote nanotube self-assembly, which is strongly pH-dependent. Basu et al. [117] used citrate-coated AuNPs to determine GSH at relatively low pH, which induces AuNP aggregation via its sulfur- and nitrogen-containing functional groups. The method proved to be very sensitive, detecting GSH at a concentration of 10^−6^ M. (iv) Inhibition of AuNPs growth induced by initial reducing agent, e.g., H_2_O_2_. The addition of antioxidants inhibits AuNPs formation in a concentration-dependent manner. An antioxidant with H_2_O_2_ scavenging activity prevents the growth of AuNPs on the SiO_2_ surface, causing a wavelength shift in the LSPR spectrum and a change in absorption intensity. Inhibition of AuNP synthesis is proportional to the antioxidant concentration and provides the basis for measuring AOxC and IC_50_, which is the antioxidant concentration that induces 50% absorption inhibition [74,75,115,116,120] (Figure 3).

(v) Surface functionalization. These mechanisms are distinguished by their selectivity, as they exploit specific interactions of a particular analyte with the functionalized nanoparticle surface. An example is the selective determination of biothiols using AuNPs nanoprobes modified with Ellman’s reagent (DTNB-Au-NP). The reaction occurring on the surface results in the release of the yellow anion 5-thio-2-nitrobenzoate (TNB^2−^), which is measured at 410 nm [29].

Bener et al. [102] proposed the use of synthesized AuNPs as a colorimetric sensor for TAC measurement. Negatively charged AuNPs stabilized with heparin adsorbed the reaction product of the CUPRAC reagent with an antioxidant, specifically the positive chelate copper(I)-neocuproine (Cu(I)-Nc) formed in the reaction of Cu(II)-Nc with antioxidants. Absorbance of the yellow-orange chromophore Cu(I)-Nc was measured at 455 nm. The authors emphasize the advantages of the developed method, i.e., resistance to aggregation, wide linear range, low LOD (0.2 µM for trolox), and higher molar absorptivity (8.36 × 10^4^ M^−1^ cm^−1^ for quercetin).

TAC can also be measured indirectly with nanoprobes through their ability to scavenge ROS/RNS. Free radicals, such as H_2_O_2_, which promote the growth of nanostructures like gold nanoshells (GNSs), are scavenged by the addition of antioxidants, thereby inhibiting this growth and attenuating the generated signal [120,121,122]. Çelik et al. [121] developed the first nanosensor using AuNPs prepared with starch for the reduction and stabilization of ss-AuNPs to evaluate the superoxide radical scavenging activity of phenolic antioxidants and to detect hydroperoxides formed during AAPH-induced oxidation of linoleic acid. The method is based on the oxidation of iodide ions by hydroperoxides to produce free iodine, followed by the subsequent formation of a triiodide ion complex. After adding the ss-AuNPs solution to the incubation mixture, triiodide ions are adsorbed on the AuNPs surface, resulting in the aggregation of gold nanoparticles. This aggregation increases the size of the ss-AuNPs from 22 nm to 75 nm, causing a red shift in the LSPR from 525 nm to 563 nm, which changes the color from red to blue-violet. Absorbance at 563 nm increases linearly with increasing hydroperoxide concentration. Phenolic antioxidants reduce the oxidation of iodide ions by superoxide radicals because they scavenge these radicals. The higher the antioxidant (scavenger) concentration, the fewer radicals are available to oxidize iodide, leading to a lower amount of triiodide ion produced and reduced aggregation of ss-AuNPs. Therefore, a reduction in SPR absorption at 563 nm allows for an indirect assessment of antioxidant activity.

Zhan et al. [123] developed a nanoprobe for determining and monitoring changes in the hydroxyl radical (OH^•^) in living cells. A probe of gold nanoclusters (AuNCs) protected by bovine serum albumin (BSA) served as a reference fluorophore, which produced an emission peak at 637 nm. A specific non-fluorescent organic dye, 2-[6-(4′-hydroxy)phenoxy-3H-xanthen-3-one-9-yl]benzoic acid (HPF), upon reaction with OH^•^, generated a product with concentration-dependent fluorescence emission at 515 nm—diaionic fluorescein—highly selective for the free radical.

Chen et al. [124] utilized unmodified gold nanoparticles (AuNPs) in the presence of single-stranded DNA (ssDNA), thereby preventing aggregation. Cleavage of ssDNA by ONOO− peroxynitrite caused the AuNPs to aggregate, and the solution color changed from red to blue. This method was used to measure the activity of the antioxidants gallic acid, ascorbic acid, and caffeic acid in scavenging ONOO−. ONOO− scavenging antioxidants restore the red color, and the decrease in absorbance is linearly dependent on the antioxidant concentration.

Lee et al. [104] developed a fluorescent probe for scavenging superoxide anion radicals (O^●−^) and OH^●^. AuNPs were modified with fluorescein-labeled hyaluronic acid (HA) capped with dopamine. The assay mechanism was based on the fragmentation of HA chains by ROS, which restores the fluorescence signal. The assay was used to evaluate the ability of other antioxidants (ascorbic acid, *p*-coumaric acid, quercetin, and α-tocopherol) to scavenge free ROS by measuring the decrease in fluorescence.

AuNPs plasmonic nanostructures can tune the shape of the nanostructure (e.g., nanoprisms, nanorods, or nanoshells) to desired optical properties and enhance LSPR sensitivity. Examples of AuNPs applications for designing effective biosensing agents are described in a review by Jain et al. [125]. Table 2 summarizes the application of AuNPs in antioxidant activity assessment, categorized by detection mechanism, type, conditions, analyzed antioxidant, and matrix.

### 3.3. Metal Oxide Nanoparticles

Several types of metal oxide nanoparticles are utilized, often due to their ability to change their oxidation state reversibly, mimicking enzymatic activity.

#### 3.3.1. Cerium Oxide Nanoparticles CeONPs or Nanoceria

AOxC determination based on CeO2NPs (CeONPs) involves the reduction of Ce(IV) ions to Ce(III) by antioxidants. The reversible oxidation state of cerium (Ce^3+^/Ce^4+^) enables the nanoparticles to change their redox states upon interaction with antioxidants [20,69,70,131]. This change in oxidation state on the NPs’ surface leads to changes in properties and color (e.g., red-orange). Measurement is performed spectrophotometrically at 510 nm or 396 nm.

CeONPs have been used as colorimetric probes on a paper platform for H_2_O_2_ detection [132]. CeONPs (NanoCerac) can be used for the rapid and sensitive detection of AOs in foods, including tea extracts, medicinal mushrooms, rapeseed, and by-products [3,69,70].

#### 3.3.2. Other Metal Oxide Nanoparticles Used for AOxC Determination

TAC tests based on Iron Oxide Nanoparticles (IONPs) involve the reduction of Fe^3+^ ions to Fe^2+^ by antioxidants in an acidic environment. The resulting Fe^2+^ is then chelated, and Fe_x_O_y_ (Fe_2_O_3_ and Fe_3_O_4_) are formed and grow at the nucleation sites, which is induced by the hydroxyl groups of the antioxidants. The formation of yellow IONP solutions is measured spectrophotometrically at 396 nm. IONPs were developed to determine the antioxidant activity of rapeseed oils at various stages of the refining process [80]. IONPs can be used to modify electrodes in electrochemical measurements of antioxidants. IONPs have high adsorption capacity and a large surface area, and their immobilization on the electrode enhances electrocatalytic oxidation [133].

Other metal oxide nanoparticles used in chemical sensing arrays include: TiO_2_ (titanium oxide), ZrO_2_ (zirconium oxide), ZnO (zinc oxide), SiO_2_ (silicon oxide), often as a carrier, NiAl_2_O_4_ (nickel aluminate) in composites for electrochemical detection, MnO (manganese oxide), and rhodium nanoparticles (RhNPs), which, when exposed to phenolic compounds, induce changes in the LSPR of rhodium nanoparticles, resulting in characteristic spectral and color transitions [15,16,18,57,66,70,71].

TiO_2_NPs are used in the electrochemical method to sensitively determine antioxidant capacity. The function of TiO_2_NPs is to generate free hydroxyl radicals through the photocatalytic oxidation of water. The antioxidant activity measurement mechanism is based on a competitive reaction, using 4-hydroxybenzoic acid (4-HBA) as the trapping agent for the OH^●^ radicals. The reaction of 4-HBA with ⋅OH radicals leads to the formation of 3,4-dihydroxybenzoic acid (3,4-DHBA). The amount of 3,4-DHBA formed is measured using square-wave voltammetry (SWV), as 3,4-DHBA and 4-HBA have different oxidation peaks (approximately 0.4 V and 0.8 V, respectively). The working electrode in this measurement is a cobalt phthalocyanine-modified screen-printed electrode (SPE). When an antioxidant is added to the system, it competes with 4-HBA for ⋅OH radical scavenging generated by TiO_2_ and UV light. Effective radical scavenging by the antioxidant inhibits the formation of 3,4-DHBA, leading to a decrease in the measured 3,4-DHBA peak current. The degree of current decrease is used to calculate the IC 50 and the Trolox equivalent (TEAC), which allows for quantification and comparison of the antioxidant capacity of the tested sample [134].

Aggregation of vanadium oxide (V_2_O_5_) nanoparticles in the presence of endogenous antioxidants Cys and GSH was used to detect them in biological media, e.g., blood, serum, and urine [135]. Aggregation of tetragonal NPs caused a color change from yellow to dark green or blue. The aggregated NPs exhibited a characteristic adsorption band at 720 nm, linearly dependent on the concentration of Cys or GSH.

Girault et al. research group [136] described the use of palladium nanoparticles deposited on a tin oxide electrode (Pd-NP-coated ITO electrodes) from an aqueous K_2_PdCl_4_ solution as a sensor to investigate the hydroxyl radical (HO^•^) scavenging capacity of antioxidants. The authors demonstrated that a catalytic reduction current results from the oxidation of freshly exposed palladium metal by OH^●^ radicals, which are generated during the reduction of oxygen or hydrogen peroxide. This mechanism was confirmed by using terephthalic acid as a fluorescent probe to scavenge OH^●^ radicals. Measurement of this catalytic current can be used to evaluate the OH radical scavenging properties of various antioxidants. When antioxidants effectively scavenge OH radicals, the number of radicals available for Pd reoxidation decreases. This, in turn, lowers the observed catalytic reduction current (IAO) (Table 3). Antioxidant activity is characterized by comparing the current in the presence and absence of AO. According to the kinetic model, there is a linear relationship between the inverse of the current difference (1/(I_c_ − I_c,AO_)) and the inverse of the antioxidant concentration (1/[AO]). The slope of the graph is inversely proportional to the OH radical scavenging rate constant (k_AO_). The smaller the slope, the higher the scavenging properties of the antioxidant.

Noteworthy is the work of Mei et al. [137], which describes a specialized nanoprobe for the selective detection of hydroxyl radicals and the evaluation of the AOxC of various compounds, such as tannic acid, ascorbic acid, and ferulic acid, as well as five traditional Chinese medicines. Luminescence from upconversion of NaYF4:Yb, Er nanoparticles was quenched initially by CA through the LRET mechanism. Later, free hydroxyl radicals formed from CA cleavage inhibited LRET, which increased luminescence. The hydroxyl radical scavenging capacity of the antioxidants was measured using luminescence imaging.

### 3.4. Nanozymes

Nanozymes are various types of nanomaterials with catalytic properties that mimic natural enzymes to directly scavenge ROS in vivo or in vitro. Nanozymes can be used not only for biosensors but also for therapeutic cytoprotective purposes. As artificial enzymes, they offer a number of advantages, the most significant of which are low cost and high stability [138].

There are two approaches to nanozyme research related to their application. The antioxidant activity of nanozymes means they are most often used in the context of therapeutic/cytoprotective applications.

For example, vanadium pentoxide (V_2_O_5_) nanowires serve primarily as GPx mimics, catalyzing the degradation of toxic H_2_O_5_ with GSH. Nanoceria mimic the action of superoxide dismutase, a component of the body’s defense against oxidative stress, helping to eliminate O_2_●^−^ and H_2_O_2_. Liu et al. [139] noted that F^−^ ions exhibit a very high affinity for nanoceria as Lewis acids and not only modify the surface of CeO_2_ nanoparticles but also accelerate electron transfer and improve the catalytic activity and stability.

However, several nanozymes are used for the detection of antioxidant molecules and the assessment of AOxC [140]. Manganese dioxide (Mn_2_O_5_) nanoplates detect GSH. Nanozymes mimic the action of peroxidase, catalyzing the oxidation of TMB to a blue product (oxTMB). The antioxidant GSH degrades the MnO_2_ nanoplates and reduces oxTMB, causing a decrease in absorbance, which is a measure of AOxC. Bimetallic gold-palladium-platinum nanorods (Au@PdPt NR) for the detection of ascorbic acid (AA). The nanozyme acts by mimicking oxidases, particularly due to the addition of Pd to the Pt nanostructure, catalyzing the oxidation of ascorbic acid (AA) in the presence of O_2_ [141]. Oxidase-mimicking cerium oxide (CeO_2_) nanoparticles were used for the colorimetric detection of AA. Graphene and carbon nanotubes, in turn, have been shown to have peroxidase-like properties, capable of catalyzing the oxidation of 3,3′,5,5′ substrates such as tetramethylbenzidine (TMB) and 2,2′-azino-bis(3-ethylbenzothiazoline-6-sulfonic acid) (ABTS) in the presence of H_2_O_2_ [138].

## 4. Quantum Dots (QDs)

Quantum dots (QDs, semiconductor nanocrystals, e.g., CdTe, CdSe) are utilized as strong fluorophores [29]. The most commonly used mechanism utilized for AOxC determination involving QDs is the “switch-on” mechanism. An example is the use of QDs to detect thiol antioxidants such as glutathione (GSH). In this case, polyaniline (PANI), which quenches the fluorescence of the QDs, is reduced by the antioxidants, restoring the QDs’ fluorescence in proportion to the antioxidant concentration [142]. Another example is the detection of polyphenols [143]. CdTe-QDs act as optical probes for the detection of polyphenols, which, upon enzymatic oxidation (e.g., by laccase) to quinones, cause fluorescence quenching. Carbon dots were considered as an ideal candidate for developing a fluorescent/colorimetric dual-mode sensor for real-time detection of the ROS, hypochlorous acid/hypochlorite (HOCl/ClO^−^), and AA as a vital antioxidant that participates in the intracellular redox homeostasis [144]. According to Wei et al. [144], RD-CDs synthesized by a one-pot hydrothermal method can respond with a fluorescent and colorimetric signal to ClO^−^ and AA immediately and reversibly.

Carbon dots (CDs) and graphene quantum dots (GQDs) are fluorescent carbon nanomaterials characterized by high biocompatibility, minimal cytotoxicity, and high sensitivity to specific biomolecules. Carbon dots (CDs/CQDs) have a discrete structure, a quasi-spherical shape < 10 nm in diameter, a nanocrystalline or amorphous structure with a cluster of sp^2^ and sp^3^ carbon with attached functional groups on their surface.

Graphene quantum dots (GQDs) are composed of single or multiple sheets of graphene < 20 nm in diameter, single- or multilayered, and most commonly hexagonal, triangular, and elliptical in shape [145].

The optical properties result from quantum confinement effects and surface states. CDs and GQDs absorb in the UV range (260–320 nm), associated with the π–π transition of C=C bonds, and occasionally a shoulder peak in the 270–390 nm region, associated with the n–π transition of C=O bonds. The addition of other atoms can alter these absorption ranges. Upon excitation, we observe strong fluorescence characterized by a high quantum yield [145].

GQDs are more attractive compared to CDs because they have a large surface area and abundant edge sides, facilitating electron transfer. Oxygen groups on the edges confer catalytic properties to GQDs.

### 4.1. Graphene Quantum Dots (GQDs)

GQDs were first described by Sun et al. in 2008 [146]. GQDs are carbon nanomaterials with a diameter smaller than 10 nm, which are non-toxic, have remarkable hydrophilicity, and have size-dependent fluorescence properties [147]. Benítez-Martínez et al. [148] used GQDs as a sensor for polyphenols, and gallic acid and oleuropein as model analytes in olive oil extracts. GQDs with an average diameter of 3.6 ± 0.9 nm, obtained by pyrolysis from citric acid, emitted blue light (474 nm) upon excitation at a wavelength of 365–420 nm. The emission maximum occurred at an excitation wavelength around 380 nm. The phenolic fraction of the extract reduced the fluorescence intensity through π–π interactions and noncovalent interactions with the GQDs. The obtained LODs were lower than 0.12 mg L^−1^, and the RSD of the results was lower than 1.7%.

El-Maghrabey et al. [149] developed a method for quantitatively determining antioxidant capacity using the fluorescence on/off properties of graphene quantum dots (GQDs). Fluorescence was quenched with Fe^3+^ ions at pH 3.5 [150]. Selected antioxidants, i.e., L-ascorbic acid, DTT, Trolox, gallic acid, pyrogallol, (+)-catechin, and caffeic acid, restored fluorescence after reduction of Fe^3+^ to Fe^2+^, depending on the antioxidant concentration. Fluorescence detection (505 nm) demonstrated improved speed and sensitivity, with a detection limit in the range of 0.603–8.23 µM for the antioxidant, compared to absorption spectrophotometry, such as FRAP. Calibration curves representing the relationship between fluorescence intensity and antioxidant concentration showed good linearity in the range of 2–100 µM, depending on the type of antioxidant, with a coefficient of determination R^2^ greater than 0.991. The test was used to visualize the distribution of antioxidants on vegetable slices (carrot, cucumber).

GQD fluorescence can be quenched using organic substances, such as dopamine (DA), which, through self-polymerization under an alkaline environment, forms a thin polydopamine (PDA) film on the quantum dot surface. The addition of antioxidants restores fluorescence by inhibiting polymerization [151]. Zhu et al. [151] used GQDs@PDA to analyze antioxidants in rat brain microdialytes.

**Figure 4:**
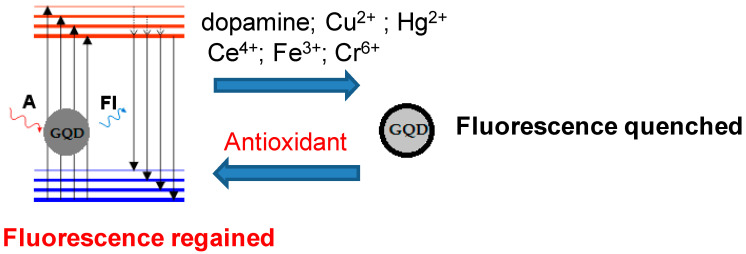
Mechanism of the antioxidant capacity assay based on fluorescence on/off of GQDs.

In other studies, fluorescence quenching of GQDs with Cu(II) was used to detect ascorbic acid (AA) and N-acetylcysteine (NAC) [152,153]; Hg^2+^ to detect GSH and Cys [154,155]; Ce^4+^, Fe^3+^, Cr^6+^, etc. to detect AA [156,157,158]. Despite the high sensitivity of these tests, it is worth noting that the use of toxic metals for GQD modification prevents their in vivo application (Figure 4).

The research group of Bhaloo et al. [159] developed a series of biocompatible structures doped with metals, including Ag-, Al-, Ce-, Fe-, Ho-, MoS_2_-, TiO_2_-, Nd-, and Tm-GQDs, which are effective in scavenging free radicals. In addition to enhancing antioxidant properties, doping GQDs can alter photoluminescence, enabling imaging of near-infrared emission.

### 4.2. Semi-Conductor Metallic Nanocrystal QDs

Semiconductor nanocrystal QDs with physical dimensions smaller than the Bohr exciton radius (2–6 nm in diameter) often consist of atoms of elements from groups II-VI (e.g., CdSe, CdTe, CdS, and ZnSe) or III-V (e.g., InP and InAs) of the periodic table [30]. However, conventional QDs are considered semiconductor structures, e.g., cadmium-based. High-quality QDs resulting from quantum confinement are characterized by a narrow, symmetric emission spectrum and resistance to photobleaching, which is why they are considered ultrasensitive fluorophores. The unique optical properties of QDs are related to their exceptional ability to tune fluorescence emission depending on the core size (size-tunable properties) and their broad excitation spectra.

In the literature, various strategies to evaluate AOxC using QDs [160,161,162,163,164,165,166,167] can be found. The use of QDs for assessing AOxC is primarily based on monitoring changes in fluorescence quenching. Antioxidants can alter the luminescence signal (fluorescence or electrochemiluminescence) of QDs through various chemical reaction mechanisms:

(i) fluorescence quenching by AOs. Fluorescence quenching occurs when antioxidants trap holes in quantum dots (QDs). The photoluminescence process in QDs begins with the excitation of an electron from the valence band to the conduction band. This excitation generates positively charged holes in the valence band and free electrons in the conduction band [18]. Once the excitation stops, the free electrons and holes recombine, resulting in the emission of a fluorescent signal. However, when antioxidants are present in the solution, they can prevent this recombination process by trapping the holes, thus quenching the fluorescence emission. It has been observed that the reduction in the fluorescence signal depends on the amount of extract added, such as *M. emarginata* plant extract or the flavonoids baicalein and hesperetin [167].

(ii) the use of electrochemiluminescence (ECL). ECL-based methods utilize QDs to detect compounds with radical scavenging functionality [160]. In the presence of QDs (e.g., CdSe) and hydrogen peroxide (H_2_O_2_), hydroxyl radicals (OH) are produced through the reduction of H_2_O_2_ by electrons injected into the QDs. Antioxidants such as glutathione (GSH) and L-Cysteine (L-Cys) scavenge these radicals, leading to the quenching effect observed in electrochemiluminescence (ECL). The ECL intensity decreases with increasing GSH and L-Cys concentrations.

(iii) Inhibition of photobleaching. This method is based on measuring the inhibitory effect of antioxidant compounds on the photobleaching process of QDs induced by UV radiation [162]. UV radiation causes a rapid decrease in the fluorescence signal of QDs (e.g., L-Cys-capped CdTe-QDs) over time—this is the photobleaching effect, caused by the generation of reactive oxygen species (ROS) catalyzed by QDs. Antioxidant compounds scavenge the generated ROS, thereby inhibiting the photobleaching effect and maintaining the fluorescence signal. The percentage of photobleaching inhibition correlates with the concentration of antioxidant compounds.

(iv) Fluorescence Restoration (Turn-on Probe). In this approach, QDs are initially quenched, and the antioxidant restores the signal [164]. The fluorescence of QDs (e.g., GSH-CdTe QDs) can be quenched by an oxidant (e.g., KMnO_4_) due to oxidation of Te atoms on the QD surface. Adding an antioxidant such as ascorbic acid (AA) reduces the oxidized forms of QDs (CdTeO_3_/TeO_2_) and restores fluorescence (a “turn-on” effect). The restored fluorescence intensity correlates well linearly with the AA concentration. Hemmateenejad et al. [164] presented an antioxidant activity assay based on the inhibition of oxidation and photobleaching of L-cysteine-capped CdTe-QDs.

QD-based methods are typically highly selective and sensitive, with excellent limits of detection (LODs). They are often used to measure the concentration of specific target antioxidants, such as GSH, Cys, or AA, in biological samples [160]. Graphene oxide (GO) can be used as an amplification platform for semiconductor quantum dots, such as CdTe QDs. The combination of graphene oxide (GO) QDs with CdTe QDs relies on the ECL quenching mechanism. GQDs amplify the signal, improving the selectivity and sensitivity of antioxidant detection, such as GSH [161]. Examples of the use of QDs for antioxidant evaluation are summarized in Table 4 depending on the mechanism of action, assay conditions and type of antioxidant.

## 5. Conclusions

Methods based on NPs for assessing AOxC utilize metallic nanoparticles (MNPs < 100 nm) and quantum dots (QDs < 10 nm) as sensitive nanosensors. These methods measure changes in Localized Surface Plasmon Resonance (LSPR) related to the formation, growth or aggregation of NPs. In contrast, QD-based AOxC assessment measures changes in fluorescence.

These assays primarily consider the total antioxidant pool in the sample, as they are non-specific to any single antioxidant class. This makes them an alternative to assays such as FC, FRAP, DPPH and ABTS. However, a few highly sensitive and specific AOxC assays utilizing modified sensors are gaining interest. These assays demonstrate selectivity for ultra-trace target antioxidants (GSH, Cys, and AA) thanks to the aggregation effect of MNPs or the quenching of QD fluorescence.

A key advantage of using NPs to assess AOxC selectively and across a broad range of antioxidants is the potential to create new assay variants depending on the sample type and analytical goals. Furthermore, both MNPs and QDs can be synthesized using green chemistry from waste products, thereby eliminating the need for complex equipment and multi-step protocols.

Despite their excellent sensitivity, which offers very good limits of detection (LOD)—crucial for measurements in biological samples—and selectivity, nanosensors require new approaches to improve stability, surface inactivation and linearity of response, especially for mechanisms based on NP formation.

Nanoparticles are undoubtedly a simple, inexpensive and highly adaptable method of measuring total AOxC. The main challenge is the lack of standardization. A significant limitation is that few nanoparticle-based AOxC assays have been compared with traditional, well-established methods such as FC, FRAP, DPPH and ABTS.

Future research should focus on developing plasmonic sensors that respond quickly at room temperature. Combining sensors with cutting-edge technologies, such as microfluidic devices, and integrating them with smartphone platforms [168], should improve reliability and accuracy. To extend the detection range of LSPR sensors beyond semiquantitative sensing, which is often susceptible to errors due to environmental factors or limited selectivity, multimodal detection techniques, including fluorescence, surface-enhanced Raman spectroscopy (SERS), and circular dichroism, should be utilized.

## Figures and Tables

**Table 1:** Summary of Nanoparticle-Based Assays for Antioxidant Capacity (AOxC).

	Assay Mechanism (Observed Change)	Detection (λmax)	Examples	Ref.
AuNPs	Formation/Growth (Reduction); Color Change: colorless salt solution to dark red/red colloidal solution.	555 nm; 540 nm; 530 nm (General LSPR for spherical AuNPs).	AOxC of phenolic acids, chrysanthemum extracts and tea beverages. Detection of Daizeol, Puerarin, and Quercetin	[77,78]
AuNPs	Aggregation Color Change: red (dispersed) to blue (aggregated).	~520 nm (for monitoring dispersed state).	Detection of polynucleotides based on distance-dependent optical properties; detection of proteins.	[65]
AgNPs	Seed Growth (SNPAC); Color Change: No color into pale yellow (seeds formation); gradual change from pale yellow to more intense in the reaction mixture (measurements)	423 nm; 405 nm (used in some comparisons).	TAC of polyphenols, vitamins C and E; Rapeseed varieties; Fruit juices and herbal teas	[73]
CeONPs (NanoCerac, CERAC)	Formation CeONPs Color Change: Formation of red-purple solutions of CeO NPs.	510 nm	TAC determination of rapeseed, white flakes, and meal	[79]
IONPs	Formation/Growth Color Change: Formation of yellow-orange solutions.	396 nm	TAC determination of rapeseed oils at various stages of the refining process.	[80]

Abbreviations: Iron Oxide Nanoparticles (IONPs); Cerium Oxide Nanoparticles (CeONPs); Silver Nano-particles (AgNPs); Gold Nano-particles (AuNPs); Antioxidants (AOs).

**Table 2:** The application of AuNPs in antioxidant activity assessment.

Detection Mechanism	Detection Type	Detection Conditions	Analyzed Antioxidant	Ref.
AuNPs Formation/Growth	Spectrophotometry (LSPR)	The absorption peaks at 555 nm	Phenolic acids (e.g., ferulic, vanillic and syringic acids) in virgin argan oil	[106]
AuNPs Formation	UV–Vis–NIR Fluorescence	The absorption peaks at ~550 nm. Fluorescence at ~448 nm (2.7 eV emission energy)	Polyphenols (59.8 mg CAE/g), terpenoids (β-cariophyllene, linalool, cis-jasmone, α-terpineol, δ-cadinene, indole, geraniol) in green tea (Camellia sinensis)	[126]
the growth of GNS precursor composites (SiO_2_/GNPs) on ITO electrode surface	the UV-vis-NIR (the intensified LSPR features); CV (reduced cathodic currents)	(SiO_2/_GNPs nanocomposites)/APTES/ITO (0.01 M PBS pH 7.4, 3.3 × 10^−4^ M AuCl_4_^−^, 1.6 × 10^−3^ M K_2_CO_3_, AOs, stirring for 30 min)	Phenolic compounds: Ferulic acid, vanillic acid, syringic acid and gallic acid	[75]
AuNPs formation	UV–vis 500–700 nm	5 mL of the sample solution, 150 µL of 0.1 M aq. HAuCl_4_ stirring and heating at 45 °C for 10 min.	The total polyphenol content of AED (153.35 ± 4.42 mg GAE g^−1^) and MED (189.79 ± 4.27 mg GAE g^−1^). The flavonoid content of AED (45.33 ± 0.14 mg QE g^−1^) and MED (49.41 ± 0.49 mg QE g^−1^) in the aqueous (AED) and methanol (MED) *Dalbergia sissoo* Roxb. extracts	[114]
AuNP enlargement, and a Au electrode modified with Au seeds	UV-vis and CV	The Au-NP growth solution: 2.06 × 10^2^ μM HAuCl_4_, 2.0 × 10^3^ μM CTAC in 1 × 10^4^ μM phosphate buffer, pH 7.0 heated for 20 min at 45 °C	Flavonoids (quercetin, daizeol and puerarin) in radix astragali, (80% flavonoids); and soybean (40% flavonoids) extracts	[111]
AuNPs growth	Spectrophotometry (LSPR). Detection at 545 nm.	100 µL of 5 × 10^−4^ M AuCl_4_^−^, 600 µL of 3.7 × 10^−3^ M CTAB, 300 µL of 2 × 10^−4^ M sodium citrate, 1 mL of extract; 10 min at 45 °C	Flavonoids, triterpenes, vitamin, and polysaccharides in (*C. morifolium*) extracts and tea beverages	[78]
GNSs as the optical nanoprobes	Spectrophotometry (the red shift from 530 nm to 780 nm)	The reduction of AuCl_4_^−^ with NaBH_4_ to GNSs used to fabrication of SiO_2_/GNPs. AOs inhibit the H_2_O_2_-induced growth of GNSs	Phenolic acids: trans-cinnamic acid, p-hydroxybenzoic acid, vanillic acid, 2,4-dihydroxybenzoic acid, protocatechuic acid and caffeic acid in licorice, mulberry leaves, chrysanthemum, green tea, black tea, honeysuckle, baicalin, ephedra and rooibos.	[120]
AuNPs growth	Spectrophotometry (LSPR) Detection at 568 nm.	Addition of 0.1 M CTAC, 0.001 M HAuCl_4_	Hydroquinone, catechol, and pyrogallol in pharmaceutical preparations and water	[112]
AuNPs growth	stopped-flow mixing technique and RLS as detection system	the presence of CTAB used as stabilizing agent	Gallic acid, propyl gallate, octyl gallate, dodecyl gallate, butylated hydroxyanisol, butylated hydroxytoluene, ascorbic acid, sodium citrate in foodstuffs	[127]
AuNPs growth	Spectrophotometry (LSPR) detection at 555 nm and CV	1 × 10^−3^ M AuCl_4_^−^, 3.7 × 10^−3^ M, CTAC, and 2 × 10^−4^ M sodium citrate in 1 × 10^−2^ M phosphate buffer (pH 8.0); 10 min heating at 45 °C	Phenolic acids: propyl gallate, caffeic acid, protocatechuic acid, ferulic acid, and vanillic acid in plant extracts and food samples	[77]
AuNPs aggregation of probes	Color change from a red-to-purple (or pink) color change) or UV-vis spectrometer	the terminal azide- and alkyne-functionalized AuNPs in the presence of Cu^2+^	AA (3 nM LOD) in citrus fruits, presence of other reducing compounds: glucose, cysteine, dopamine, thiamine and uric acid	[118]
AuNPs precursor composite (SiO_2_)/AuNP)	SERS	AOs inhibit Au^3+^ reduction in the presence of H_2_O_2_ and deposition of Au^0^ onto the surface of the SiO_2_/AuNPs.	tannic acid, citric acid, ferulic acid, and tartaric acid	[74]
Au nanozyme-sensor	CV, DPV, EI	AuNPs nanozymes act as natural peroxidases (e.g., horseradish peroxidase)	lavender and sea buckthorn extracts; Trolox as standard AO	[103]
growth of AuNPs precursor composite (SiO_2_)/AuNP)	UV–vis spectrophotometry, SERS	SiO_2_/AuNPs, K_2_CO_3_/HAuCl_4_ solution, AOs were stirred for 30 min at RT	phenolic acids: vanillic acid (10–250 µM), syringic acid (10–110 µM), and gallic acid (5–55 µM)	[128]
Au@CuS core–shell loaded with CuO_2_ NPs	SERS (decrease in the Raman signal of OXTMB)	a slightly acidic environment, Cu^2+^, H_2_O_2_, TMB, GSH	GSH; LOD 1.2 × 10^−13^ mol∙L^−1^ in serum samples	[129]
AuNPs growth	image acquisition using a desktop flatbed scanner; visual or optical color changes on paper based sensor	paper nucleation of AuNPs as colorimetric probes	GAE in teas and wines Linear range: 10 μM–1.0 mM, LOD < 1.0 μM, 3.6–12.6% RSD	[130]
AuNPs Growth	The absorbance at 537, 539, 571, 573 nm.	0.01 M phosphate buffer (pH 8.0), 15.2 µM CTAC, 1 mM HAuCl_4_, AOs; stirred (2 min), heated at 45 °C (10 min), frozen 25 min	aglycones-genistein, daidzein, and glycosides: genistin and daidzin in soy extracts	[113]

Abbreviations: cetyltrimethylammonium bromide (CTAB); the resonance light-scattering (RLS); Gold nanoshells (GNSs); indium tin oxide (ITO); limit of detection (LOD); Surface-enhanced Raman spectroscopy (SERS); tetramethylbenzidine (TMB); tetramethylbiphenyl (OXTMB); glutathione (GSH); cetyltrimethylammonium chloride (CTAC); Room Temperature (RT); γ-Aminopropyltriethoxysilane (APTES); chlorogenic acid equivalents (CAE); Gold nanoshells (GNSs); three-dimensionally (3D); room temperature (RT); Gallic acid equivalents (GAE).

**Table 3:** Key chemical/electrochemical processes and reactions in the kinetic model for the determination of antioxidant activity using Pd-NP-coated ITO electrodes according to Girault et al. [136].

Process	Reaction	Rate Constant	Mechanism
Active Site Generation (Electrochemistry)	PdO + H_2_O + 2e→Pd + 2OH^−●^	*k*_0_	Electrochemical reduction of PdO to metallic Pd, exposing active sites
OH Radical Generation (Catalytic)	H_2_O_2_→2OH^−●^	*k*_1_	Catalytic dissociation rate constant of H_2_O_2_ on the freshly exposed metallic Pd
H_2_O_2_ Generation (In Situ)	O_2_ + H_2_O + 2e→H_2_O_2_ + 2OH^−●^	*k_r_*	Electrochemical reduction of dissolved O_2_ to H_2_O_2_
Catalytic Cycle (Current Enhancement)	Pd + 2OH^−●^→PdO + H_2_O	*k*_2_	Re-oxidation: OH^−●^ reoxidize metallic Pd to PdO causing the enhancement of the cathodic reduction current
Competitive Consumption (AO Measurement)	OH^−●^ + AO→H_2_O + AO^+^ (AO^+^ oxidized form of AO)	*k_AO_*	Scavenging: AO competes with Pd for the OH^−●^. Higher *k_AO_* results in a lower catalytic current
Additional Reactions	H_2_O_2_ + 2e→2OH^−●^	*k*_3_	Electrochemical reduction rate constant of H_2_O_2_

**Table 4:** Application of QDs for AOxC assessment.

Mechanism	QDs	Target/Type of Antioxidant (LODs)	Measurement Conditions	Ref.
ECL Quenching	CdSe QDs	GSH (1.0 μM), L-Cys (2.0 μM)	OH^●^ are produced from H_2_O_2_ reduction by electron-injected QDs; AOs scavenge free radicals, causing ECL quenching.	[160]
ECL Quenching—Scavenging of CdTe-QDs^●+^ amplified by Graphene Oxide (GO)	CdTe QDs (amplified by GO)	GSH (8.3 μM)	Selective detection of GSH due to stronger hydrogen bonding with GO than GSSG or Cys. GO enhances the ECL signal and generation of QD radicals CdTe-QDs^●+^ GSH causes total quenching of ECL.	[161]
Fluorescence Restoration	GSH-CdTe QDs (capped by GSH)	AA (74 nM) in urine, plasma	QDs are initially quenched by KMnO_4_ (oxidation of Te atoms). AA reduces the oxidized forms (CdTeO_3_/TeO_2_), restoring the fluorescence (turn-on effect).	[162]
Photoluminescence Quenching	CdS/dendrimers	AA (3.3 μM) in tablets	AA quenches the photoluminescence, which is linearly correlated with AA concentration.	[163]
Inhibition of Photobleaching	CdTe-QDs (L-Cys)	Flavonoids (quercetin, tannic acid, caffeic acid, gallic acid, naringin, trolox); in teas	the QD solution (30 nM) generate ROS under UV irradiation (254 nm) for 30 s, causing photobleaching. AOs scavenge ROS, inhibiting bleaching and preserving fluorescence. AOxC is measured as percentage inhibition of photobleaching.	[164]
Fluorescence Quenching	TGA-capped CdTe-QDs	Extract Merremia emarginata (Polyphenols) in herbs	AOs trap the holes (positive charge carriers) created during excitation, preventing electron-hole recombination and thus quenching the fluorescent emission. The reduction in the signal depends on the amount of extract added.	[165]
Fluorescence Quenching	CdTe QDs	Baicalein (24.5 ngmL^−1^; 1.3%RSD), Hesperitin (9.7 ng mL^−1^; 1.97%RSD) in urine	Quenching due to optical and electrochemical interaction. Conditions: Tris-HCl buffer, pH 7.4, 0.24 mM QDs, 10 min of incubation	[167]

Abbreviations: electrochemiluminescence (ECL); glutathione (GSH); L-Cysteine (L-Cys); antioxidants (AOs); Ascorbic acid (AA); Thioglycolic acid (TGA).

## Data Availability

No new data were created or analyzed in this study.

## Abbreviations

The following abbreviations are used in this manuscript:

3,4-DHBA	3,4-dihydroxybenzoic acid
4-HBA	4-hydroxybenzoic acid
A	absorbance
AA	Ascorbic acid
AAPH	2,2′-Azobis(2-amidinopropane) dihydrochloride
ABTS	2,2′-azinobis(3-ethylbenzthiazoline-6-sulfonic acid) assay
AFM	atomic force microscopy
AgNPs	silver nanoparticles
AOs	Antioxidants
AOxC	antioxidant capacity
AuNCs	gold nanoclusters
AuNPs	gold nanoparticles
BSA	bovine serum albumin
CA	carminic acid
CDs	Carbon dots
CE	Capillary Electrophoresis
CeONPs	cerium oxide nanoparticles
CTAB	cetyltrimethylammonium bromide
CTAC	cetyltrimethylammonium chloride
Cu(I)-Nc	copper(I)-neocuproine
CUPRAC	Cupric Reducing Antioxidant Capacity
CV	cyclic voltammetry
Cys	cysteine
DLS	Dynamic Light Scattering
DPPH	2,2-diphenyl-1-picrylhydrazyl
DPV	differential pulse voltammetry
DTNB	Ellman’s reagent: 5,5′-Dithio-bis(2-nitrobenzoic acid)
ECL	Electrochemiluminescence
EM	Electromagnetic field
EPR	Electron paramagnetic resonance spectroscopy
ET	single electron transfer
FC	Folin–Ciocalteu method
FIA-AD	flow injection analysis with amperometric detection
FRAP	Ferric Reducing Antioxidant Power Assay
FTIR	Fourier transform infrared spectroscopy
GC	Gas Chromatography
GNSs	gold nanoshells
GO	Graphene Oxide
GQDs	graphene quantum dots
GSH	glutathione
GSSG	glutathione disulfide
HA	hyaluronic acid
HAT	hydrogen atom transfer
HPF	2-[6-(4′-hydroxy)phenoxy-3H-xanthen-3-one-9-yl]benzoic acid
HPLC	high-performance liquid chromatography
IC50	Inhibitory Concentration 50%
IONPs	Iron Oxide Nano-particles
ITO	indium tin oxide
LOD	limit of detection
LRET	luminescence resonance energy transfer
LSPR	Localized Surface Plasmon Resonance
Mel	melamine
Met	methionine
MNPs	metal nanoparticles
NPs	Nanoparticles
ORAC	Oxygen radical antioxidant capacity
OXTMB	tetramethylbiphenyl
PBN	N-tert-butyl-α-phenylnitrone
QDs	quantum dots
RhNPs	rhodium nanoparticles
RLS	the resonance light-scattering
RNS	reactive nitrogen species
ROS	Reactive Oxygen Species
SEF	Surface-enhanced fluorescence
SEIRA	Surface-enhanced infrared absorption
SEM	Scanning electron microscopy
SES	Surface-enhanced spectroscopies
SERS	Surface-enhanced Raman spectroscopy
SNAPC	Silver NanoParticle Antioxidant Capacity
SP	surface plasmons
SPE	screen-printed electrode
SPR	surface plasmon resonance
ss-AuNPs	starch-stabilized gold nanoparticles
ssDNA	single-stranded DNA
SWV	square-wave voltammetry
TAC	Total antioxidant capacity
TEAC	trolox equivalent antioxidant capacity
TEM	transmission electron microscopy
TEMPOL	4-hydroxy-2,2,6,6-tetramethyl-1-piperidinyloxyl
TMB	tetramethylbenzidine
TP	total phenolic compounds
TRAP	total peroxyl radical-trapping antioxidant parameter assay
UV–Vis	Ultraviolet-visible (UV-vis) spectroscopy
XRD	X-ray diffraction
